# Observations on the Use of Celandine (Chelidonium) in the Venereal Disease

**Published:** 1804-02-01

**Authors:** 

**Affiliations:** Erlangen


### OBSERVATIONS *on the* USE *of* CELANDINE *(Chelidonium) in the* VENEREAL DISEASE, *by Professor* WENDT, *of Erlangen.*

THE expectations of medical men have been so frequently frustrated, in respect to the discovery of a medicine for the venereal disease, which might serve as a sure substitute for mercury, without being attended with the disagreeable consequences often observed after the continued use of mercurials, that the introduction of any new remedy to accomplish the much desired purpose has always been received with prejudice. However, the results of experience, relative to this subject, are extremely contradictory; and while some physicians assert they have cured many venereal patients by the sole use of vegetable and similar remedies, the observations of others are calculated entirely to discredit them; a fate which the famous decoction of Guajar, so greatly praised by an early writer on the venereal disease, Chevalier de Hutten, has shared with Astragalus exscapus, Clematis erecta and vitalbæ, Sarsaparilla, Dulcamara, Carex arenia, Nitric acid, &c. Although I cannot account for these results, yet they appear to me to prove, that no certain evidence is given for or against the use of such remedies, which may be considered as decisive on either side; it might therefore, even from this circumstance, be excusable in venturing to publish another; the more so, if it be approved by experience and the evidence of facts.

The remedy I allude to is the common Celandine (Chelidonium majus, L.) which plant has long been celebrated in several disorders, and which I know, from my own practice, to be an excellent medicine in obstinate agues, obstructions of the liver, jaundice, exanthemata, &c. I had, however, never employed it in the venereal disease till I was accidentally informed that Dr. Hechtel, of Bayreuth, made an extensive use of it in the cure of that disorder. Accordingly, I gave the Celandine a trial by employing it in about twenty cases, wherein I succeeded far beyond my expectation. Although I was greatly importuned to make the remedy known to the public, I withheld the discovery for the following reasons; First, because the cases in which I had successfully employed it, did not seem to me wholly sufficient for ascertaining the fact; Secondly, because I imagined my patients might have used mercurials before the time they applied to me, or even during during the period they were under my care; an imposition which not unfrequently happens to physicians; and, Thirdly, because I did not think myself entitled to make public a remedy which I knew was kept secret by Dr. Hechtel, without having previously obtained his permission. I therefore wrote to the Doctor, and acquainted him with the means by which I had procured information respecting his remedy; I also related to him the successful use I had made of it, and intimated to him that it would be of infinite utility were the medicine published for the benefit of the community. In a few days after the Doctor returned the following answer.

"I have used that remedy (celandine) longer than eight years, during which time it has done me the greatest service in more than fifty cases, some of which were so obstinate, as even to withstand the effect of mercurials. I order pills, of two grains each, to be made of the extractum chelidonii and the radix chelidonii, and I prescribe my patients two in the morning, and two at night, increasing the dose by degrees from twenty to thirty, which are taken twice pro dosi; afterwards I gradually diminish the dose in the same manner I had increased it. In some of my patients the remedy would cause a diarrhœa, which I arrested by discontinuing the medicine for some days; while, in others, it produced profuse sweats, a symptom I always disliked, as it considerably retarded the cure. I shall be glad if you will undertake the publication of a remedy, which is approved by your and my own experience."

Much, however, as I rely on the evidence of so able a practitioner, I am far from thinking his remedy to be infallible, yet from the success with which it has been given, both by him and by me, it seems to be deserving of farther trials, and it would afford me particular satisfaction, if I could persuade medical men to examine this remedy in their practice, and afterwards to communicate the results, whether successful or unsuccessful, of its internal application.

In summer I generally give the juice of the radix and herba (succus expressus) with honey, and diluted with water; first, in the dose of a tea-spoon, gradually increasing it to a table spoonful, which dose I order to be continued till the cure is finished. In autumn and spring I employ the succus expressus radicis chelidonii; and in winter, the extract, which is generally made of the whole plant. But as most persons dislike taking expressed juices, I generally give the following pills:

℞. Succi

R. Succi recenter express. hb. et rad. chelidonii majoris aa. unc. iij. inspiss. leni caloris gradu ad consistent mellis. dein adde pulver. radic. chelidon. major. q. v. satis ad consistentiam massæ pilularis; ex qua formentur pil. pond. gr. ij. consper. pulver. lycopodii. serv. usui.

Of these pills I order two to be taken in the morning and evening, daily increasing the dose by one pill, till I have raised the dose to ten pills, which I continue till the cure is completed. I have often employed the herb and the root, each separately, and the latter appeared to me more efficacious. If it be intended to quicken the cure, I prescribe the pills to be taken every three hours. I have not yet observed any symptom, which could prejudice the use of this remedy, as diarrhœas, sweats, or salivation, but only found that in some cases it diminishes the appetite, which however is easily remedied by stomachics.

Case I. A stout labourer, aged thirty-seven, applied to me for assistance. As he complained of violent pain in the right testicle, which was extremely swoln ; the pains extended to the crista ilei and the perinæum, and the glandulæ inguinales were likewise swoln and painful ; I immediately ordered a suspensorium to be applied, and gave him the pills in the manner I have mentioned. The first four or five days no abatement in the pains and swelling could be observed ; but after this time both complaints considerably decreased, and a gonorrhœa supervening, the pains and swelling almost entirely ceased. Having continued the remedy about three weeks, he recovered so far that he could again follow his business.

Case II. The wife of the last mentioned patient, who had most probably been infected by her husband, complained of a burning sensation which she felt on discharging her urine, of pains in the throat, and of hoarseness. On examining her, I found several ulcers behind the tonsils; I ordered her to gargle with water and honey, and gave the pills of Chelidonium. When she had continued them about five weeks, she found herself cured.

Case III. A boy, fourteen years of age, who was born of venereal patients, complained of pains in the inguinal region, and some time after a swelling was observed in the right side, on which a surgeon applied a plaster ; but the swelling and pains increasing, the tumour broke, and then formed an ulcer, on which the emplastrum diachylon simplex plex was put. When he was brought to me, I found the ulcer very foul, and discovered similar ulcers in the fauces. He was directed to gargle with honey and water, the ulcer in the inguen to be dressed with four parts of unguent. dig. off. and one part of unguentum Ægypticum; and internally he took the pills of chelidonium. By this treatment he was cured in the space of four weeks; but having afterwards caught a violent cold, which threatened a relapse, he had recourse to the pills, and was entirely freed from his complaints.

Case iv. With the same success I cured a young man of a phymosis, applying at the same time some external remedies, particularly the balsamus sulphuris, prepared with oleum lini, with which the swelling of the prepuce was dressed, and I injected likewise a decoction of scordium between the glans and the prepuce.

Case v. A young man, aged nineteen, being infected with the venereal disease, committed himself to my care. After the disease had lasted for ten days, during which time he had used a decoction of sarsaparilla and graminis without any avail, I found three ulcers on the prepuce, one of which had eaten very deeply, and several complaints were observed in the fauces; the tonsils and uvula were swoln, and covered with a yellow purulent matter. In order to try the effects of chelidonium I employed no mercurials, but prescribed the following pills,

℞. Extr. chelidon. maj. dr. jß, f. pil. ponder. gr. ij. consp. pulver. hb. chelidon. D. S. two pills to be taken in the morning and at night.

For gargling I recommended oxymel simplex with water, and the ulcers I covered with dry lint. Three days after he got much better, the complaints in the fauces had entirely ceased, the ulcers of the prepuce had a better appearance, and were cleaner; but about this time the patient having heated himself with wine, had a great deal of pain in the inguinal glands, which began to swell. I applied on them pieces of linen spread with extractum chelidonii, and ordered six pills to be taken every day, by which means I not only removed this new complaint, but the ulcers were cured in less than three weeks. The patient however continued the medicine a fortnight longer, and was then completely cured.

Case vi. A young man, 23 years of age, who two years ago had been afflicted with complete lues, in which he was treated treated by a barber, retained a purulent running of the urethra, which was extremely troublesome. I prescribed him three pills per day of the chelidonium, which having continued some days the running began to diminish; but as it again increased, I raised the doses from four to six, whereby the gleet gradually decreased; after some weeks it entirely ceased, and has never since returned.

Case VII. A girl, eighteen years of age, had every symptom of venereal infection, difficulty of swallowing, swelling of the palate, pains in the urethra, &c. I accordingly prescribed, Extr. chelidon, dr. ij. pulv. cort. Peruv. q. s. ut f. pil. pond. gr. ij. consp. pulv. cinnamonni d. s. six pills to be taken every day, and gradually to increase the dose. When she had used this medicine for about a fortnight, all the venereal symptoms gradually disappeared, and the patient finding herself recovered, could not be prevailed upon to continue the medicine any longer.

Case VIII. Another woman complained of the same symptoms as the last mentioned patient, though in a less degree. I prescribed as follows: Extr. chelidon. dr. ij. taraxaci dr. ß. pulv. cort. Peruvian. q. s. pilul. ponder. gr. ij. consperg. pulv. cinnam. I ordered her at first to take six pills a day, but by degrees to increase the dose to twelve, and she had hardly consumed the prescribed quantity, when she found herself free from all the venereal symptoms.

In all these cases I employed no other medicine than the chelidonium, and it surprized me therefore, when I met with several cases in which I gave this efficacious remedy without effect. It so far discouraged me, that I gave up all thoughts of farther employing it in the venereal disorder. Considering, however, that the ill success which happened to me in several cases might be owing to the inordinate use of that medicine, which patients of this kind are very apt to, and to the disorderly manner of living so frequently observable in venereal subjects, but particularly at the request of some of my friends, I again had recourse to that remedy. I also determined to make a trial of the chelidonium glaucium, L. or yellow-horned poppy, a plant frequently cultivated in our gardens; and I had the satisfaction of curing several cases without the assistance of any other medicine; in some, however, I used mercurials externally. I either gave the fresh succus expressus of the root root and herb in the dose of a tea spoonful every three hours, or in form of extract; viz. Extr. chelidon. glaucii q. l. pulver. glycirrhiz. q. s. ut f. pil. ponder. gr. ij. involve pulv. fab. d. s. two pills to be taken every three hours. This dose I gradually increased, as circumstances allowed; it would sometimes cause vomiting and diarrhœa, in which case I diminished the dose. For gargling and fomentations in phymosis and paraphymosis, and for washing the ulcers and warts, I applied the juice of the same plant or the extract, both diluted with water.

Case I. A young woman was affected with venereal ulcers in the fauces, and ulcers and warts of the genitals, which even extended to the anus; at the same time she had a very malignant fluor albus. After having used the chelidonium glaucium, in the manner just stated, internally and externally, for about three weeks, the ulcers began to heal, and the warts to dry and to fall off; the fluor albus diminished and became less corrosive.

Case II. With the same success I employed the chelidonium glaucium in a case of phymosis, and chancres of the genitals.

Case III. A young man was afflicted with a very bad paraphymosis, in which the glans was swollen to an enormous degree; the opening of the urethra was so compressed that not even the smallest bougie or catheter could be passed, I therefore proposed the operation for paraphymosis, with which however the patient would not comply. I then ordered the parts to be fomented with warm milk, and by degrees with diluted extract of chelidonium, and gave him the pills, by which treatment the patient gradually recovered in the course of five weeks.

Case IV. A gentleman, aged fifty-six, had frequently exposed himself in his youth to venereal infections, after which he had retained a hardness and tumour of the right testicle, a curvature of the urethra, and a gleet, which frequently returned. These circumstances however did not prevent him from marrying and having several children; and though these symptoms could not be considered as venereal, he wished to get rid of them. I accordingly gave him the pills of chelidonium glaucium, increasing the medicine till he took ten at each dose, which after some time completely relieved him from those complaints.

